# Germline ERCC excision repair 6 like 2 ( *ERCC6L2* ) mutations lead to impaired erythropoiesis and reshaping of the bone marrow microenvironment

**DOI:** 10.1111/bjh.18466

**Published:** 2022-09-26

**Authors:** Hannah Armes, Findlay Bewicke‐Copley, Ana Rio‐Machin, Doriana Di Bella, Céline Philippe, Anna Wozniak, Hemanth Tummala, Jun Wang, Teresa Ezponda, Felipe Prosper, Inderjeet Dokal, Tom Vulliamy, Outi Kilpivaara, Ulla Wartiovaara‐Kautto, Jude Fitzgibbon, Kevin Rouault‐Pierre

**Affiliations:** ^1^ Centre for Genomics and Computational Biology Barts Cancer Institute, Queen Mary University of London London UK; ^2^ Centre for Haemato‐Oncology Barts Cancer Institute, Queen Mary University of London London UK; ^3^ Centre for Genomics and Child Health Blizard Institute, Queen Mary University of London London UK; ^4^ Área de Hemato‐Oncología CIMA Universidad de Navarra, Instituto de Investigación Sanitaria de Navarra (IDISNA), Centro de Investigación Biomédica en Red de Cáncer, CIBERONC Pamplona Spain; ^5^ Clínica Universidad de Navarra Pamplona Spain; ^6^ Applied Tumor Genomics Research Program, Faculty of Medicine University of Helsinki Helsinki Finland; ^7^ HUSLAB Laboratory of Genetics, HUS Diagnostic Center Helsinki University Hospital Helsinki Finland; ^8^ Department of Medical and Clinical Genetics, Medicum, Faculty of Medicine University of Helsinki Helsinki Finland; ^9^ Department of Hematology Helsinki University Hospital Comprehensive Cancer Center Helsinki Finland

**Keywords:** acute myeloid leukaemia (AML) and myelodysplastic syndrome (MDS), familial leukaemia, haematopoietic stem/progenitor cells, mesenchymal cells, niche and bone marrow microenvironment

## Abstract

Despite the inclusion of inherited myeloid malignancies as a separate entity in the World Health Organization Classification, many established predisposing loci continue to lack functional characterization. While germline mutations in the DNA repair factor ERCC excision repair 6 like 2 (*ERCC6L2*) give rise to bone marrow failure and acute myeloid leukaemia, their consequences on normal haematopoiesis remain unclear. To functionally characterise the dual impact of germline *ERCC6L2* loss on human primary haematopoietic stem/progenitor cells (HSPCs) and mesenchymal stromal cells (MSCs), we challenged *ERCC6L2*‐silenced and patient‐derived cells *ex vivo*. Here, we show for the first time that *ERCC6L2*‐deficiency in HSPCs significantly impedes their clonogenic potential and leads to delayed erythroid differentiation. This observation was confirmed by CIBERSORTx RNA‐sequencing deconvolution performed on *ERCC6L2*‐silenced erythroid‐committed cells, which demonstrated higher proportions of polychromatic erythroblasts and reduced orthochromatic erythroblasts versus controls. In parallel, we demonstrate that the consequences of *ERCC6L2*‐deficiency are not limited to HSPCs, as we observe a striking phenotype in patient‐derived and *ERCC6L2‐*silenced MSCs, which exhibit enhanced osteogenesis and suppressed adipogenesis. Altogether, our study introduces a valuable surrogate model to study the impact of inherited myeloid mutations and highlights the importance of accounting for the influence of germline mutations in HSPCs and their microenvironment.

## INTRODUCTION

Germline mutations predisposing to myeloid malignancies have been identified in >20 genes and while some are biologically well annotated, others remain poorly characterised and are emerging from basic research.^1^, ^2^ Knowledge of the causative lesion in familial disease is critical for optimal management of patients and their families, and presents a unique opportunity to gain insights into the aetiology of myeloid malignancies, distinct from the study of acquired mutations.

Homozygous germline loss‐of‐function ERCC excision repair 6 like 2 (*ERCC6L2*) variants were first reported in two unrelated cases of bone marrow failure (BMF) with developmental delay and microcephaly.^3^ Subsequently, germline *ERCC6L2* variants have been detected in 2%–4% of individuals with a personal or family history suggestive of inherited myeloid disease.^1^, ^4^, ^5^ These patients typically present with BMF and ~30% develop myelodysplastic syndrome (MDS) or acute myeloid leukaemia (AML). A specific *ERCC6L2* variant, (NM_020207.7) c.1424del: p.Ile475ThrfsTer36, reported in 12 patients and enriched in the Finnish population (Genome Aggregation Database [gnomAD] V3.1.2 allele frequency: 6.03 × 10^−3^) is directly associated with development of erythroid leukaemia – a rare and aggressive subset of AML accounting for 3%–5% of sporadic cases.^6^

Functionally, ERCC6L2 contributes to the transcription‐coupled nucleotide excision repair pathway, with mutated cells demonstrating increased sensitivity to DNA‐damaging agents.^3^, ^7^ While recent knockout mouse models support a role for Ercc6l2 in non‐homologous end‐joining facilitating B‐cell isotype switching,^8^ the role of ERCC6L2 in haematopoiesis remains unknown. Furthermore, it is still unclear whether the effects of germline mutations are restricted to the haematopoietic compartment alone, or if changes in the bone marrow stroma also contribute to disease initiation and progression.

Here, we show for the first time that *ERCC6L2*‐deficient cells bear considerable phenotypic changes within both the haematopoietic and stromal compartments. We observe significant loss of haematopoietic stem progenitor cell (HSPC) clonogenic potential and delayed erythropoiesis, in addition to extensive lineage skewing in mesenchymal stromal cells (MSCs) and confirm that our *ex vivo* model successfully recapitulates the phenotype of patient cells.

## EXPERIMENTAL PROCEDURES

For a more detailed description of the methods used, see the Online Supplemental Information.

### Source of primary human HSPCs and MSCs

Umbilical cord blood (UCB) samples were purchased from Anthony Nolan (London, UK). Bone marrow (BM) aspirates were obtained after formal consent from healthy young adult donors from CIMA Universidad de Navarra (Navarra, Spain). Patient samples, P1 and P2, were sourced after informed consent from Helsinki University Hospital Comprehensive Cancer Center (Helsinki, Finland). Healthy donor BM samples were age‐matched to the patient samples. Details of patient samples are provided in Supplemental Table S1.

### Generation of lentiviral vectors and viral particles

In all, 21 base pair lentiviral short‐hairpin RNA (shRNA) vectors targeting *ERCC6L2* and non‐target Scramble control were purchased from VectorBuilder Inc. (Chicago, IL, USA). Target sequences and reporter genes are summarised in Supplemental Table S2. Viral particles for all shRNAs were produced by transient CaCl_2_ transfection of HEK293‐T cells. At 48 h after transfection, virus was harvested by ultracentrifugation at 54 000 *g* for 2 h at 4°C. Viral titres were determined using HEK293‐T cells and measured by flow cytometry based on enhanced green fluorescent protein (EGFP) expression.

### Cell purification and transduction of HSPCs

Mononuclear cells (MNCs) were isolated from UCB or BM by centrifugation using Ficoll‐Paque PLUS (GE Healthcare Life Sciences) followed by red blood cell lysis. CD34^+^ cell selection was performed using the EasySep™ Human CD34 Positive Selection Kit II (StemCell Technologies) according to the manufacturer's instructions. CD34^+^ cells were stimulated using StemSpan medium (StemCell Technologies) supplemented with cytokines (150 ng/ml stem cell factor [SCF], 150 ng/ml FMS‐like tyrosine kinase‐3 ligand [Flt3‐L], 10 ng/ml interleukin 6, 25 ng/ml granulocyte colony‐stimulating factor, 20 ng/ml thrombopoietin [TPO]; PeproTech) and 1% hydroxyethylpiperazine‐ethane‐sulphonic acid buffer (HEPES; Sigma‐Aldrich) for 4–6 h. Virus particles were added to the stimulated cells at a multiplicity of infection (MOI) of 30 and incubated at 37°C, 5% CO_2_ overnight. Cells were washed and re‐suspended in expansion medium (StemSpan with 150 ng/ml SCF, 150 ng/ml FlT3‐ligand, 20 ng/ml TPO; PeproTech) and expanded for 4 days prior to fluorescence‐activated cell sorting.

### Culture of primary HSPCs

The HSPCs were cultured in expansion media (StemSpan with 150 ng/ml SCF, 150 ng/ml FlT3‐ligand, 20 ng/ml TPO; PeproTech) for 14 days and media was changed every 3–4 days. Cell number was measured at day 7 and 14 using a Countess 3 Automated Cell Counter (ThermoFisher) and cells were isolated for RNA at day 0, 10 and 14. Erythropoiesis was induced by culturing HSPCs in erythroid differentiation medium (25 ng/ml SCF, 3 U/ml erythropoietin, 50 ng/ml insulin‐like growth factor‐1; PeproTech) for 14 days. Cells were immunophenotyped by flow cytometry at day 3, 7, 10 and 14. Cell number was measured at day 7 and 14 and cells were isolated for RNA at day 14. Granulopoiesis was induced by culturing HSPCs in granulocytic differentiation media (25 ng/ml SCF, 10 ng/ml granulocyte‐macrophage colony‐stimulating factor; Peprotech) for 14 days.

## RESULTS

### Silencing of *ERCC6L2* reduces HSPC clonogenic potential

**FIGURE 1:**
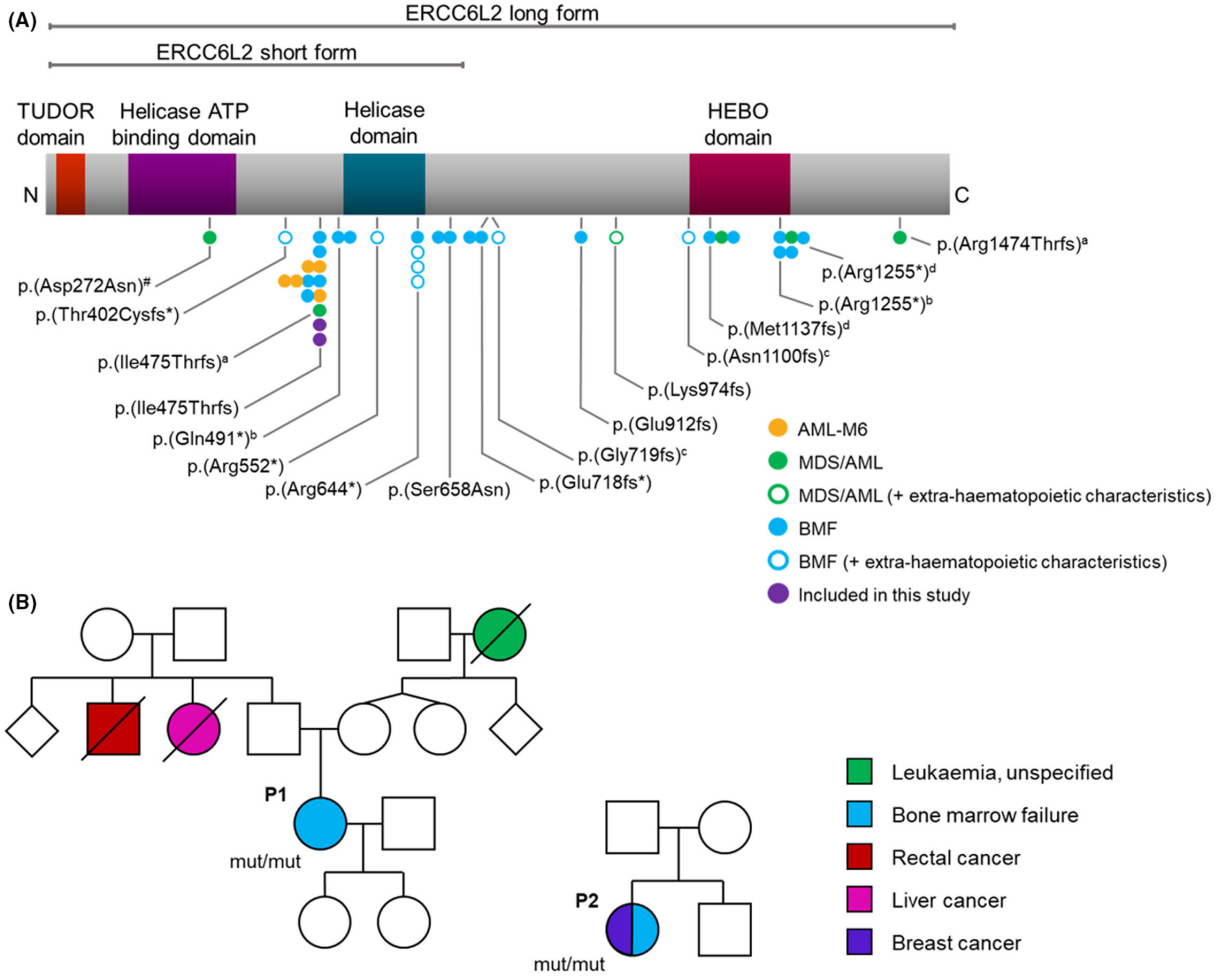
Germline *ERCC6L2* variants in patients with BMF, MDS and AML. (A) Schematic of the *ERCC6L2* transcript (NM_020207.7) and its protein domains with the location of reported *ERCC6L2* mutations (*N* = 32). Circles represent individual cases and are colour‐coded by disease manifestation; circles connected horizontally are affected individuals from the same pedigree. Patients included in this study are represented in purple. ‘Extra‐haematopoietic characteristics’ refers to the presence of developmental delay, learning difficulties and/or microcephaly. ^a,b,c,d^Mutations are all homozygous aside from four compound heterozygotes where the co‐occurring *ERCC6L2* mutations are indicated with a superscript letter. ^#^Patient also had a germline telomerase reverse transcriptase (*TERT*) mutation. (B) Schematic representation of families included in this study. Patients, P1 and P2, are indicated. Both patients harboured germline homozygous *ERCC6L2* mutations (p.Ile475ThrfsTer36). P1 presented with BMF during childhood and experienced several thrombocytopenic episodes in early adulthood, while P2 was diagnosed with mild cytopenia, BM hypoplasia and breast cancer at 36 years. P1 has previously been published with patient reference 1438.^6^ AML, acute myeloid leukaemia; BMF, bone marrow failure; ERCC6L2, ERCC excision repair 6 like 2; MDS, myelodysplastic syndrome.

The DNA repair protein ERCC6L2 exists as multiple isoforms including a short form (SF; 701 amino acids) containing the catalytic ATP/Helicase domain, and a canonical long form (LF; 1550 amino acids) containing the HEBO domain, found to be responsible for the homing of ERCC6L2 to sites of damaged chromatin. Both ATP/Helicase and HEBO domains are essential for protein function.^8^, ^9^ Germline *ERCC6L2* variants are distributed along the entirety of the gene, almost exclusively lead to frameshifts or truncations and have been shown to result in loss of gene activity (Figure 1A).^3^ *ERCC6L2* is ubiquitously expressed in haematopoietic cells and MSCs, although its expression varies during differentiation (Figure S1).

**FIGURE 2:**
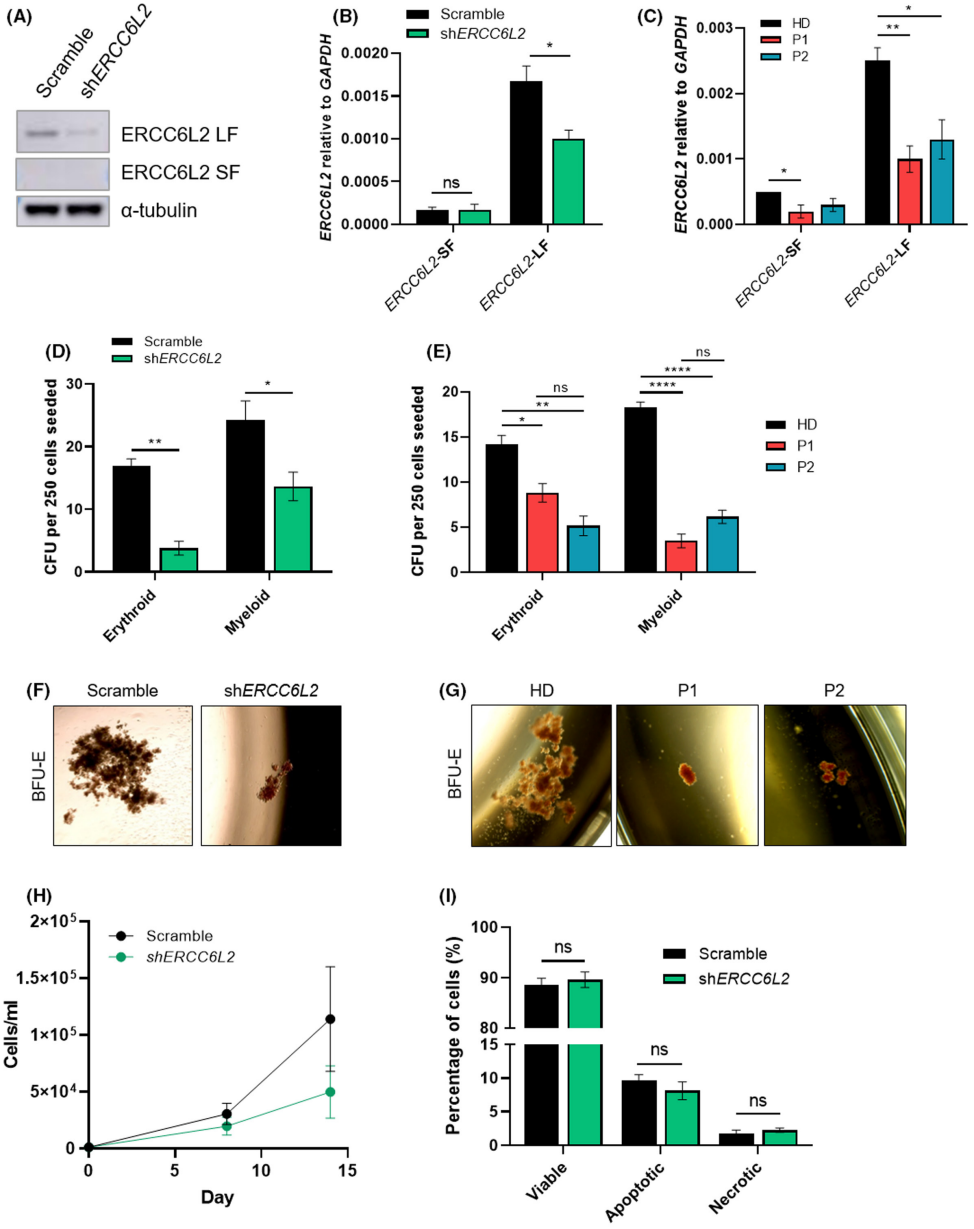
*ERCC6L2*‐silencing in HSPCs leads to reduced clonogenic potential. (A) ERCC6L2 protein expression in scramble and *ERCC6L2*‐KD HSPCs grown in erythroid media for 14 days, analysed by Western blotting. (B) RT‐qPCR assessment of *ERCC6L2* short‐form (SF) and long‐form (LF) expression relative to *GAPDH* in *ERCC6L2*‐KD and scramble HSPCs. Data represents three biological repeats. (C) RT‐qPCR assessment of *ERCC6L2*‐SF and *ERCC6L2‐*LF expression relative to *GAPDH* in patient and healthy donor HSPCs. (D) Colony forming unit (CFU) assay of CD34^+^ cells derived from healthy donor umbilical cord blood (UCB) and transduced with *ERCC6L2‐*KD or scramble control. Colonies are separated by CFU type. Data represents three biological repeats. (E) CFU assay of CD34^+^ cells derived from BM of two unrelated patients (P1 and P2) with homozygous germline *ERCC6L2* mutations, compared with healthy donor (HD) BM controls. Colonies are separated by CFU type. Data represents three technical repeats. (F) Representative images of morphology of burst forming unit‐erythroid (BFU‐E) colonies derived from scramble or *ERCC6L2*‐KD CD34^+^ cells. Images were captured at ×4 magnification. (G) Representative images of morphology of BFU‐E colonies derived from patient or HD CD34^+^ cells. Images were captured at ×4 magnification. (H) Expansion rate of *ERCC6L2*‐KD and scramble HSPCs grown in expansion media across 14 days. Data represents three biological repeats. (F) Percentages of viable, annexin‐V^+^ apoptotic and DAPI^+^ necrotic HSPCs. *ERCC6L2*‐KD and scramble HSPCs were grown in expansion media and analysed after 3 days of culture. Data represents three biological repeats. Results are shown as the mean ± SEM. *p < 0.05; **p < 0.01; ****p < 0.0001. BM, bone marrow; DAPI, 4',6‐diamidino‐2‐phenylindole; ERCC6L2, ERCC excision repair 6 like 2; GAPDH, glyceraldehyde 3‐phosphate dehydrogenase; HSPC, haematopoietic stem progenitor cell; KD, knockdown; RT‐qPCR, reverse transcription quantitative polymerase chain reaction.

To investigate the impact of loss of *ERCC6L2* expression on normal haematopoiesis, we assessed the effect of *ERCC6L2*‐silencing in HSPCs (CD34^+^) using a knockdown (KD) strategy. HSPCs isolated from UCB were transduced with a lentiviral‐shRNA vector, Scramble or sh*ERCC6L2*, prior to cell sorting based on expression of an EGFP reporter gene 4 days after transduction. KD efficacy of two shRNAs was initially tested in the OCI‐AML3 cell line (Figure S2) followed by phenotypic analysis in HSPCs to confirm off‐target effects. The shRNA eliciting the most significant KD in *ERCC6L2* (namely, sh*ERCC6L2‐*#1*)* was then taken forward for all downstream experiments. HSPCs derived from the BM MNCs of two patients (P1 and P2) with germline *ERCC6L2* mutations were isolated and cultured in parallel with the KDs for functional and transcriptomic profiling. Both patients harboured identical germline homozygous *ERCC6L2* mutations (p.Ile475ThrfsTer36) with two or more acquired mutations in tumour protein p53 (*TP53*). P1 presented with BMF during childhood and experienced several episodes of mild thrombocytopenia in early adulthood, in contrast to P2 who was diagnosed with mild cytopenia, BM hypoplasia and breast cancer at 36 years (Figure 1B; Table S1). KD and patient HSPCs both exhibited significant reductions in *ERCC6L2* expression, specifically affecting the canonical LF of the gene (Figure 2A–C).

As patients with *ERCC6L2* mutations are predisposed to myeloid disorders, we postulated that *ERCC6L2‐*silencing would impede myeloid differentiation. Firstly, we examined the effect of *ERCC6L2‐*KD on HSPC clonogenic capacity by performing colony‐forming assays, which revealed a significant reduction in colony formation in both *ERCC6L2*‐KD and patient HSPCs (Figure 2D,E). The erythroid lineage appeared to be particularly affected, with *ERCC6L2*‐deficient erythroblasts forming significantly fewer and markedly smaller burst forming unit‐erythroid (BFU‐E) colonies (Figure 2F,G). Although HSPCs were less proliferative, no increase in apoptosis or marked changes to the cell cycle were detected (Figure 2H,I, Figure S3A,B). These experiments were confirmed in parallel studies using the second shRNA (Figure S4A–H). Overall, these findings suggest our *ERCC6L2*‐KD model faithfully recapitulates the phenotype of patient HSPCs and that *ERCC6L2* loss significantly impedes HSPC clonogenic potential.

### Loss of *ERCC6L2* in HSPCs delays erythropoiesis

**FIGURE 3:**
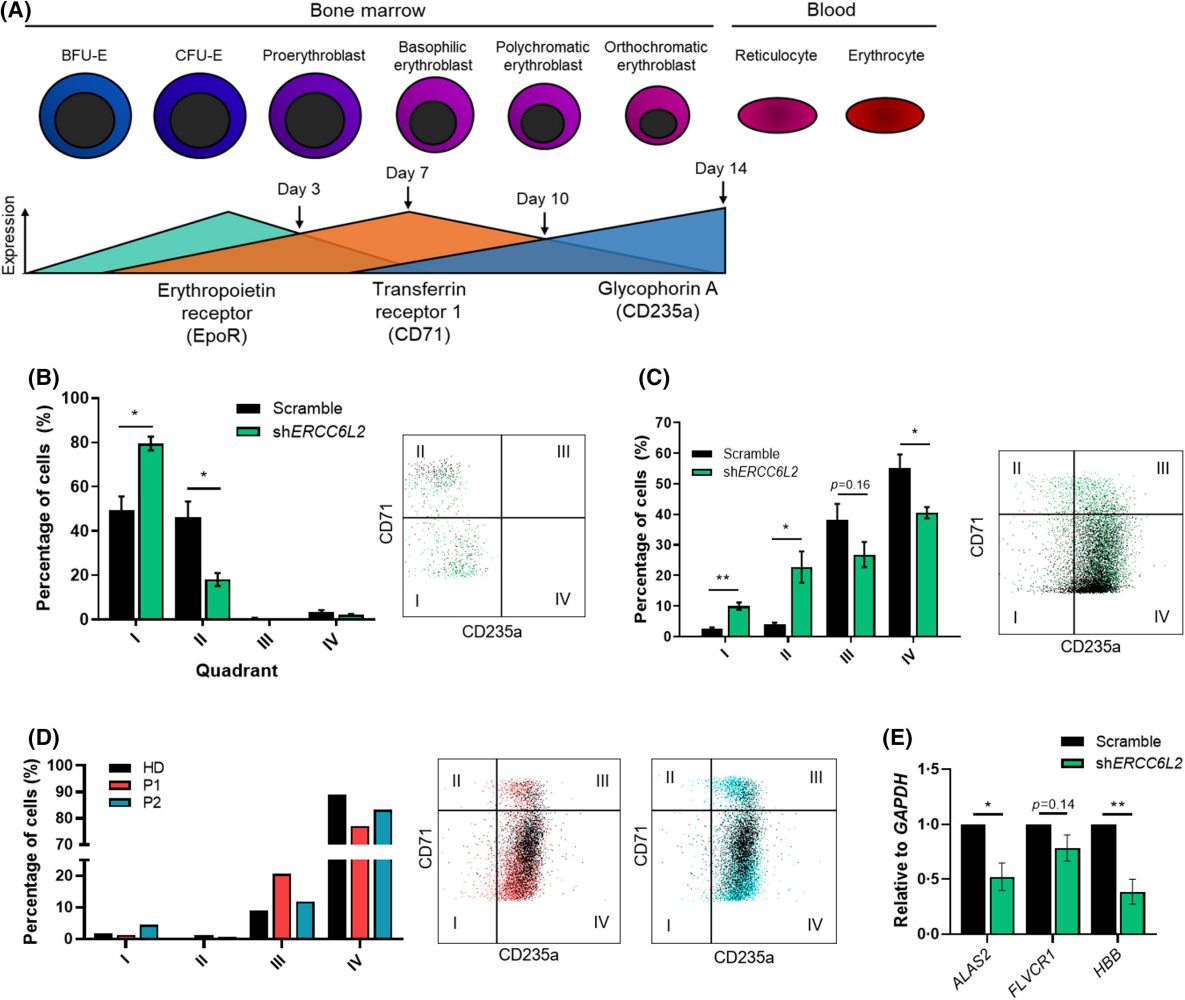
*ERCC6L2*‐deficient erythroid‐committed HSPCs are delayed in their capacity to differentiate. (A) Schematic of erythroid differentiation, adapted from Yang et al. 2016.^32^ During normal erythropoiesis, pro‐erythroblasts initially show an upregulation of transferrin receptor 1 (CD71) that is progressively lost with a concomitant increase in glycophorin a (CD235a) as they mature. (B) Erythroid immunophenotyping analysis in *ERCC6L2*‐KD and scramble CD34^+^ cells at day 3. Representative flow plot is shown. Data represents three biological repeats. (C) Immunophenotyping analysis of CD71 and CD235a expression in scramble or *ERCC6L2*‐KD CD34^+^ cells cultured in erythroid media for 14 days. Data represents three biological repeats. Representative flow plot is shown. (D) Erythroid immunophenotyping analysis in patient‐derived and healthy donor CD34^+^ cells at day 14. Representative flow plots are shown. (E) RT‐qPCR analysis of erythroid marker genes at day 14 relative to *GAPDH* in *ERCC6L2*‐KD and scramble HSPCs. Expression of genes of interest are normalised to scramble. Results are shown as the mean ± SEM. *p < 0.05; **p < 0.01. ERCC6L2, ERCC excision repair 6 like 2; GAPDH, glyceraldehyde 3‐phosphate dehydrogenase; HSPC, haematopoietic stem progenitor cell; KD, knockdown; RT‐qPCR, reverse transcription quantitative polymerase chain reaction.

Considering the prevalence of erythroid leukaemia in patients and the observed clonogenic phenotype, we next sought to measure the impact of *ERCC6L2‐*silencing on erythropoiesis. The HSPCs were cultured in erythroid differentiation conditions for 14 days and immunophenotyped by flow cytometry every 3–4 days. During normal erythropoiesis, pro‐erythroblasts initially show an upregulation of transferrin receptor 1 (CD71) at days 3–7 that is progressively lost as the cells differentiate from day 10–14, with a concomitant increase in glycophorin A (CD235a) expression as the cells mature, peaking at day 14 (Figure 3A).

In line with the reduced BFU‐E clonogenicity, *ERCC6L2*‐KD HSPCs displayed delayed erythroid differentiation as they were slower to acquire CD71^+^ by day 3 (Figure 3B), while at day 14 fewer *ERCC6L2*‐KD cells expressed markers of late‐stage differentiation (CD71^−^/CD235a^+^) versus controls (Figure 3C). Patient HSPCs displayed a similar phenotype, with fewer mature cells present at day 14 and a higher proportion of cells retaining CD71^+^ expression compared to healthy donor HSPCs (Figure 3D). Moreover, reverse transcription quantitative polymerase chain reaction (RT‐qPCR) analysis of key genes in erythropoiesis (5'‐aminolevulinate synthase 2 [*ALAS2*], the enzyme essential for initiation of heme synthesis; FLVCR haem transporter 1 [*FLVCR1*], an exporter of excess haem to avoid toxicity; haemoglobin subunit beta [*HBB*], a haemoglobin subunit) revealed a significant downregulation of *ALAS2* and *HBB* in the *ERCC6L2*‐KD erythroid cells, suggesting that haem and globin synthesis were suppressed (Figure 3E) and indicative of ineffective erythropoiesis. Altogether, this analysis suggests that loss of *ERCC6L2* expression impedes erythroid differentiation.

**FIGURE 4:**
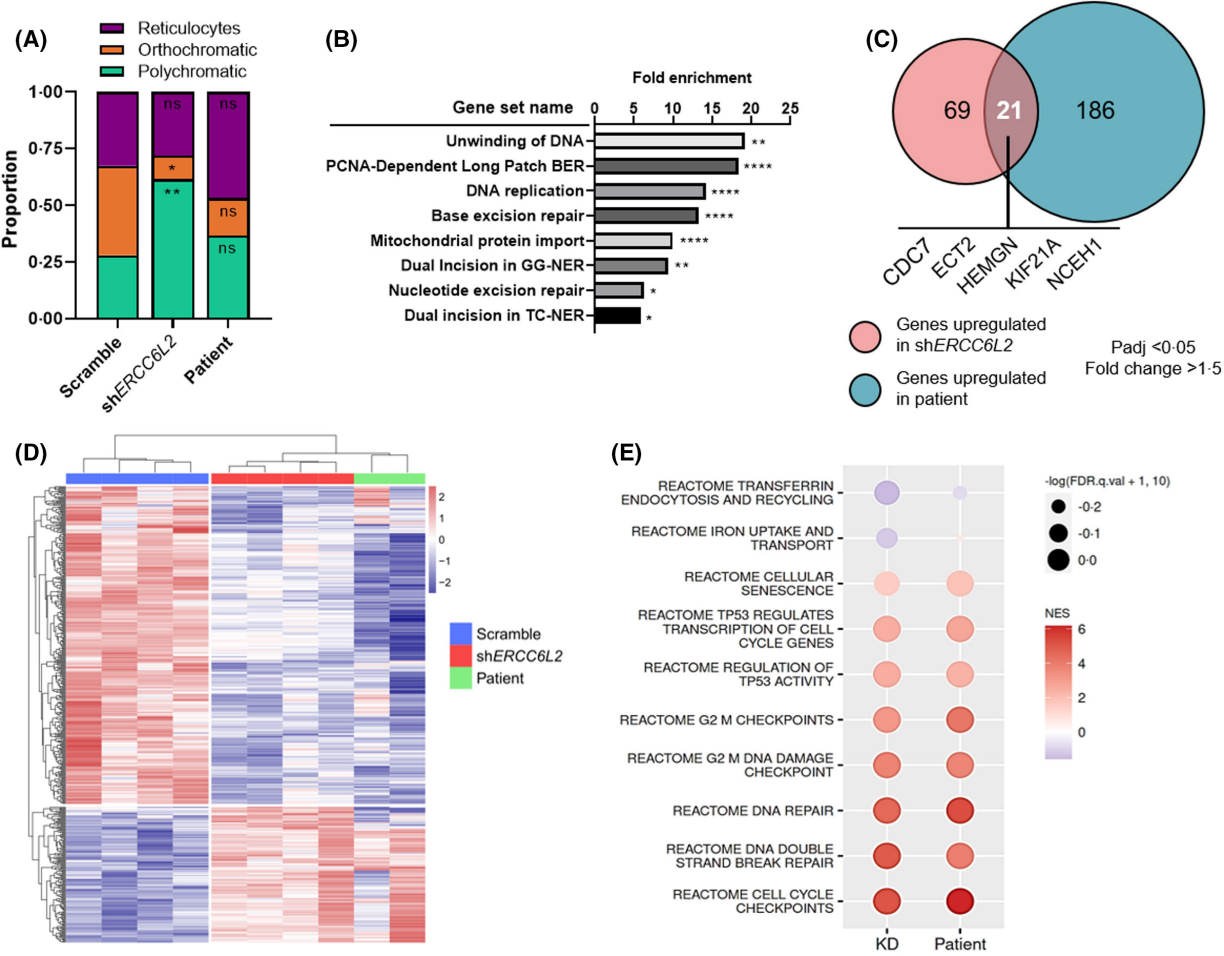
Transcriptomic signatures are shared between *ERCC6L2*‐KD and patient erythroid‐committed HSPCs. (A) *In silico* deconvolution analysis inferring the mean relative abundance of erythroid cell types within scramble, *ERCC6L2*‐KD and patient‐derived HSPC populations, which had been grown in erythroid media for 14 days and analysed by bulk RNA‐seq. Significance values represent KD or patient cells versus scramble control. (B) Functional annotation of the genes in the scRNA‐seq reticulocyte signature as determined by DAVID. Upregulation is presented as fold enrichment. (C) Venn diagram showing genes that were significantly upregulated in *ERCC6L2*‐KD and patient‐derived HSPCs compared to scramble, *p*adj < 0.05, fold change >1.5. HSPCs were grown in erythroid media for 14 days. (D) Heat map showing hierarchical clustering of differentially expressed genes (DEGs) in *ERCC6L2*‐KD (*N* = 4, sh*ERCC6L2*‐#1) and scramble (*N* = 4) compared to patient‐derived (*N* = 2) HSPCs, all cultured in erythroid media for 14 days. (E) Dot plot of gene set enrichment analysis (GSEA) showing positive or negative normalised enrichment scores (NES) in *ERCC6L2*‐KD or patient‐derived HSPCs compared to scramble HSPCs grown in erythroid media for 14 days (FDR <0.25). Results are shown as the mean ± SEM. *p < 0.05; **p < 0.01; ****p < 0.0001. BER, base excision repair; ERCC6L2, ERCC excision repair 6 like 2; FDR, false discovery rate; GG‐NER, global genomic nucleotide excision repair; HSPC, haematopoietic stem progenitor cell; KD, knockdown; PCNA, proliferating cell nuclear antigen; TC‐NER, transcription‐coupled nucleotide excision repair.

To substantiate our findings, we generated RNA‐sequencing data from *ERCC6L2*‐deficient erythroid‐committed HSPCs and used an *in silico* approach to infer cell abundance at various stages of differentiation within our populations. Based on gene signatures derived from single‐cell RNA‐sequencing analysis of erythroid subtypes,^10^, ^11^ we performed CIBERSORTx deconvolution on our bulk RNA‐sequencing data sets. Our analysis revealed that *ERCC6L2‐*KD and patient erythroid cells had higher proportions of polychromatic erythroblasts compared to Scramble (61.5% and 36.6% respectively), which were enriched instead for more mature subtypes, orthochromatic erythroblasts (39.4%) or reticulocytes (32.6%) (Figure 4A; Figure S5A). Giemsa staining of erythroid‐committed HSPCs at day 14 showed a similar trend, with fewer mature erythroblasts/reticulocytes observed in the KD population (Figure S5B). Furthermore, within the reticulocyte signature, we observed an over‐representation of genes associated with excision repair (Figure 4B) suggesting that functional DNA repair pathways may be a requirement for terminal differentiation. Overall, both the *in silico* analysis and immunophenotyping suggest that *ERCC6L2‐*deficiency results in delayed erythropoiesis.

### *ERCC6L2‐*silencing in HSPCs leads to upregulation of DNA repair and TP53 activity pathways

Next, we performed differential gene expression and gene set enrichment analysis (GSEA) on *ERCC6L2*‐deficient erythroid‐committed HSPCs to examine the molecular pathways impacted by *ERCC6L2‐*silencing. In all, 21 genes were significantly upregulated in both patient and *ERCC6L2*‐KD cells (Figure 4C; *p*adj < 0.05, fold change >1.5), including genes involved in DNA damage recovery (cell division cycle 7 [*CDC7*],^12^ epithelial cell transforming 2 [*ECT2*]^13^) and regulation of erythropoiesis (hemogen [*HEMGN*]^14^, ^15^). Moreover, hierarchical clustering of differentially expressed genes showed that patient and KD cells clustered most closely together (Figure 4D), suggesting that *ERCC6L2*‐deficiency drives a specific biological signature.

Moreover, we observed a marked similarity in the gene sets significantly up‐ and down‐regulated in KD and patient cells compared to Scramble (Figure 4E). Prominently, KD cells showed down‐regulation of the ‘transferrin endocytosis and recycling’ gene set, supporting our observation that transferrin receptor 1 (CD71) expression is altered with *ERCC6L2*‐silencing. Furthermore, multiple DNA repair pathways were significantly upregulated in the KD and patient cells spanning both single‐ and double‐strand break responses, accompanied by enrichment of several TP53 activity and cell cycle checkpoint gene sets. Altogether, our analysis indicated that *ERCC6L2‐*deficiency leads to suppression of gene sets involved in haematopoietic differentiation, while DNA repair and TP53 activity pathways are significantly upregulated.

### Patient MSCs exhibit lineage skewing

The BM niche plays a key role in sustaining haematopoiesis by balancing HSC self‐renewal and differentiation,^16^ but it can also contribute to development and propagation of myeloid diseases.^17^, ^18^, ^19^ Germline variants offer a unique opportunity to study the dual role of mutations in the haematopoietic and stromal compartments. To investigate the effect of *ERCC6L2* loss in the BM, MSCs isolated from BM MNCs of healthy donors were transduced with the same lentiviral shRNA used in our HSPCs (sh*ERCC6L2*‐#1), and the resultant KD MSCs were cultured in parallel with MSCs isolated from BM MNCs of patients (P1 and P2). Both *ERCC6L2*‐KD and patient MSCs demonstrated a significant reduction in *ERCC6L2* expression compared to controls (Figure S6A–C).

**FIGURE 5:**
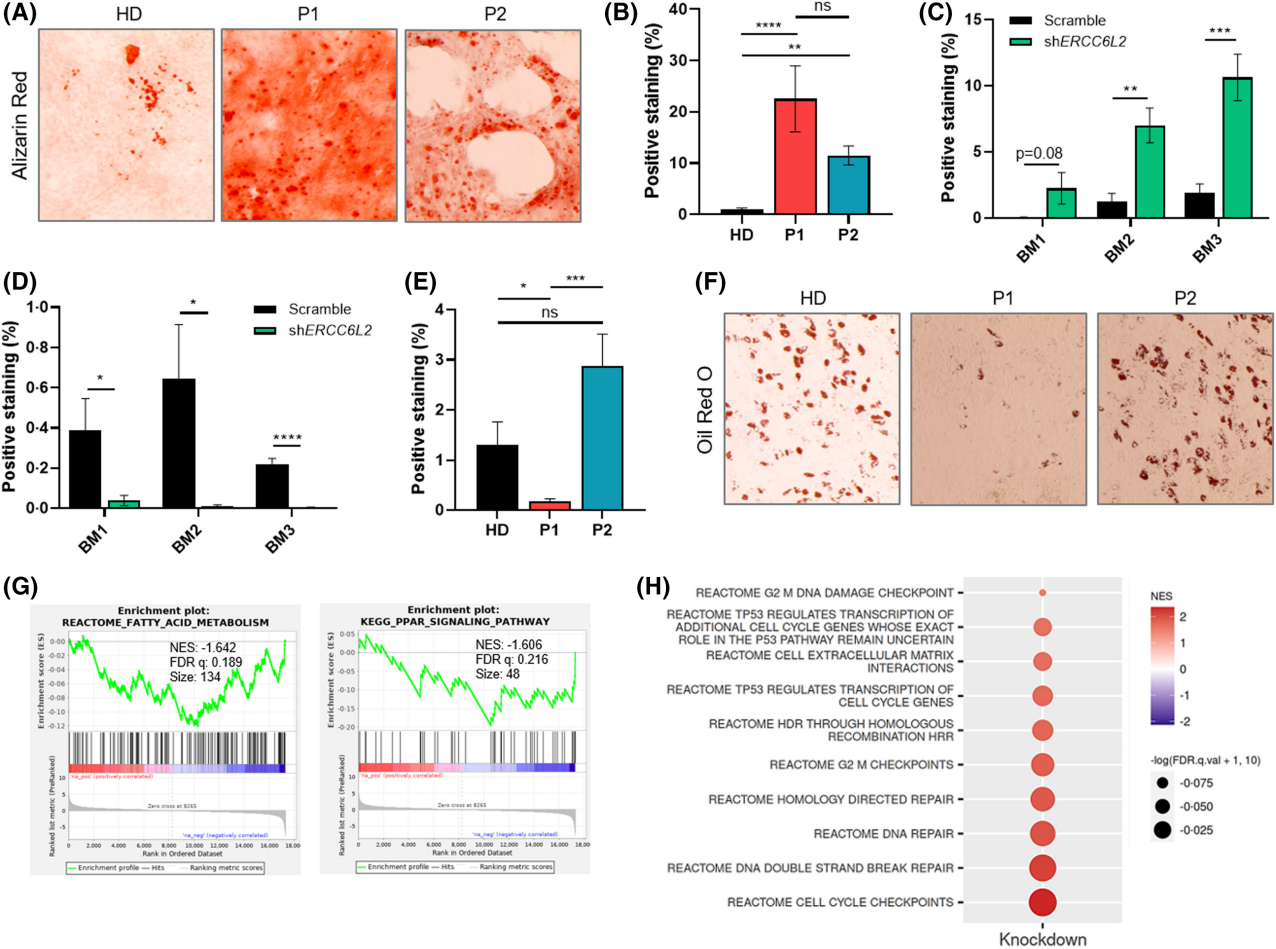
*ERCC6L2‐*deficient MSCs show a lineage skewing. (A) Representative images of Alizarin Red staining in patient and healthy donor (HD) MSCs cultured in osteogenic differentiation media for 14 days. (B) Quantification of Alizarin Red staining in patient and HD MSCs cultured in osteogenic differentiation media for 14 days. Area of positive staining from eight fields of view was quantified using ImageJ software. (C) Quantification of Alizarin Red staining in scramble and *ERCC6L2‐*KD MSCs cultured in osteogenic differentiation media for 21 days. Area of positive staining from eight fields of view was quantified using ImageJ software. Figure shows three biological repeats. (D) Quantification of Oil Red O staining in scramble and *ERCC6L2‐*KD MSCs cultured in adipogenic differentiation media for 21 days. Area of positive staining from eight fields of view was quantified using ImageJ software. Figure shows three biological repeats. (E) Quantification of Oil Red O staining in patient and HD MSCs cultured in adipogenic differentiation media for 14 days. Area of positive staining from eight fields of view was quantified using ImageJ software. (F) Representative images of Oil Red O staining in patient and HD MSCs cultured in adipogenic differentiation media for 14 days. (G) GSEA plots showing downregulation of fatty acid metabolism and PPAR signalling gene sets in *ERCC6L2*‐KD MSCs compared to scramble when cultured in adipogenic media for 21 days. (H) Dot plot of GSEA showing normalised enrichment scores (NES) in *ERCC6L2*‐KD MSCs compared to scramble MSCs, grown in expansion media for 21 days (FDR <0.25). Results are shown as the mean ± SEM. *p < 0.05; **p < 0.01; ***p < 0.001; ****p < 0.0001. ERCC6L2, ERCC excision repair 6 like 2; FDR, false discovery rate; GSEA; gene set enrichment analysis; KD, knockdown; MSC, mesenchymal stromal cell; PPAR, peroxisome proliferator‐activated receptor.

As MSCs give rise to essential niche‐supporting cells including osteoblasts and adipocytes,^20^ we explored whether *ERCC6L2‐*deficiency would affect differentiation into these lineages. MSCs were cultured in osteogenic or adipogenic differentiation conditions prior to staining and quantification. Strikingly, *ERCC6L2*‐KD and patient MSCs cultured in osteogenic media presented a significant increase in Alizarin Red staining compared to controls, representing greater deposition of calcified matrix (Figure 5A–C, Figure S7A). The cells also developed morphological hallmarks of osteoblasts, e.g., gap junctions and pseudopodia, at earlier time‐points, as observed in images captured at day 14 (Figure S7B), supporting the notion that enhanced osteogenesis accompanies *ERCC6L2‐*silencing.

Conversely, when *ERCC6L2*‐KD MSCs were cultured under adipogenic conditions and stained with Oil Red O to visualise intracellular fat deposits they showed a significant reduction in staining compared to controls, suggesting that adipogenesis was disrupted (Figure 5D, Figure S7C). The phenotype of MSCs cultured in adipogenic media was more variable between the two patients, as P1 had significantly less staining than the healthy donor, indicative of fewer fat deposits, while P2 had similar staining to the control (Figure 5E,F), although it is possible that the normal cellular composition of the BM of P2 may have been disrupted by their recent chemotherapy. Overall, these data demonstrate that loss of *ERCC6L2* impacts the BM niche cells by consistently enhancing osteogenesis while also potentially modulating adipogenesis.

To determine whether *ERCC6L2‐*deficiency in MSCs could affect their supportive capacities, we performed a long‐term co‐culture assay with wild‐type HSPCs seeded on *ERCC6L2‐*KD, patient, or control MSCs (Figure S8A). After 5 weeks, the haematopoietic cells were harvested and re‐plated for colony‐forming assays. Under these conditions, co‐culture of HSPCs and *ERCC6L2*‐deficient MSCs had no significant effect on the number of colonies that formed (Figure S8B–D).

### *ERCC6L2‐*silencing enhances osteogenic potential and suppresses adipogenic pathways

We next sought to determine which transcriptional programmes were dysregulated due to *ERCC6L2‐*silencing in MSCs using the RNA‐sequencing approach previously described. When analysing the transcriptomes of *ERCC6L2‐*deficient MSCs we observed the dysregulation of several genes/pathways consistent with phenotypes observed in culture. In *ERCC6L2*‐KD osteoblasts, bone morphogenetic protein 2 (*BMP2*), a potent inducer of osteogenesis, was enriched, while adipogenic markers (complement factor D [*CFD*]; adiponectin, C1Q and collagen domain containing [*ADIPOQ*]) and gene sets (fatty acid metabolism, peroxisome proliferator‐activated receptor [PPAR] signalling) were downregulated in *ERCC6L2‐*KD adipocytes (Figure 5G, Figure S9A,B). These trends were confirmed in patient cells by RT‐qPCR, where we found *BMP2* and *BMP4* to be significantly upregulated in osteoblasts derived from both patients (Figure S9C), while adipogenic markers PPAR gamma (*PPARG*), *CFD* and *ADIPOQ* were upregulated in P2 but downregulated in P1 (Figure S9D) consistent with the contrasting adipogenic phenotypes observed in our cell culture assays. Moreover, GSEA analysis of *ERCC6L2*‐deficient MSCs identified enrichment of pathways previously identified in HSPCs, including DNA repair and TP53 activity, reinforcing the notion that specific gene sets related to *ERCC6L2* loss are impacted across diverse cell types (Figure 5H).

## DISCUSSION

Germline mutations in *ERCC6L2* have been convincingly linked to development of BMF and MDS/AML, with >30 patients now reported in the literature,^3^, ^4^, ^5^, ^6^, ^7^, ^9^, ^21^, ^22^ and while some molecular hallmarks of *ERCC6L2‐*deficiency have been described,^3^, ^7^ the implications on haematopoiesis are ill‐defined. Here, using a combination of *ex vivo* cell culture and transcriptomic analysis, we demonstrate that *ERCC6L2‐*deficiency in KD and patient‐derived HSPCs results in significantly impaired clonogenicity. Importantly, our model also suggests that *ERCC6L2‐*deficient HSPCs experience delayed erythropoiesis. It is possible that the loss of *ERCC6L2* induces a stress on erythroid progenitors that fosters the acquisition of secondary mutations and contributes to the erythroid leukaemia phenotype observed in *ERCC6L2‐*deficient patients.

While our understanding of *ERCC6L2*‐mediated predisposition to myeloid disease is still evolving, the link between impaired DNA damage repair factors and inherited BMF syndromes is already well established and drives the phenotype of Fanconi anaemia (FA) and DNA Ligase IV Deficiency syndrome.^23^, ^24^ Biallelic mutations in *ERCC6L2* and *FANC* genes result in similar biological features: FA patient cells are prone to DNA damage and consequently exhibit hyper‐activated TP53, triggering cell cycle arrest.^24^, ^25^, ^26^ Similarly, our group have previously shown that *ERCC6L2*‐deficient patient‐derived lymphoblastoid cell lines are significantly more sensitive to DNA damaging agents compared to controls, with irofulven treatment leading to accumulation of DNA damage marker, 53BP1, and increased cell cycle arrest at G2/M phase.^27^ In our present study, our transcriptomic analysis further supports enrichment of these pathways, highlighting a high level of TP53 activity and cell cycle checkpoint gene sets in *ERCC6L2*‐silenced HSPCs and MSCs.

While the phenotypic consequences of *ERCC6L2‐*deficiency in our haematopoietic cells were considerable, mounting evidence indicates that germline mutations can induce significant dysregulation of the BM microenvironment. Here we sought to investigate the consequences of *ERCC6L2‐*silencing in the stromal niche and resultantly observed a lineage skewing in KD and patient‐derived MSCs, with enhanced osteogenesis and suppressed adipogenesis. While this phenotype differs from an archetypal BMF where the BM would be considered unregenerative, adipogenic and impaired in its capacity to undergo osteogenesis, it is instead reminiscent of sporadic AML, where the adipocytic niche becomes compromised, resulting in suppressed normal myelo‐erythropoiesis, permitting blasts to thrive.^28^, ^29^ This semblance may suggest that patients harbouring *ERCC6L2* mutations possess a microenvironment primed for malignancy. While our co‐culture assays did not reveal defects in the capacity of *ERCC6L2*‐deficient MSCs to support normal HSPC growth/renewal, there is an unmet need for development of more complex *ex vivo* models in order to reveal the full multidimensional impact of germline mutations on normal haematopoiesis.

In summary, there is a now a much greater appreciation of germline mutations in myeloid malignancies, including their contribution to the aetiology of disease and the overlap or uniqueness to sporadic MDS/AML.^30^, ^31^ Here, we have developed a model that reliably recapitulates the key features of the germline *ERCC6L2* phenotype, including reduced clonogenicity, delayed erythropoiesis and lineage skewing in the BM microenvironment. Overall, we show for the first time the impact of *ERCC6L2‐*deficiency in HSPCs and their microenvironment and demonstrate that our KD system can be used as a surrogate model to study the impact of other inherited mutations on the haematopoietic and stromal compartments.

## AUTHOR CONTRIBUTIONS

Jude Fitzgibbon, Kevin Rouault‐Pierre and Hannah Armes conceived the project, designed the experiments and wrote the manuscript. Hannah Armes performed the experiments. Ana Rio‐Machin, Anna Wozniak, Doriana Di Bella and Céline Philippe helped with data collection and some experiments. Findlay Bewicke‐Copley and Jun Wang performed RNA sequencing data analysis. Felipe Prosper and Teresa Ezponda contributed healthy donor BM samples and provided single‐cell RNA sequencing data. Outi Kilpivaara and Ulla Wartiovaara‐Kautto contributed patient samples. All co‐authors reviewed the manuscript.

## CONFLICT OF INTEREST

The authors declare no competing financial interests.

## Supporting information


Appendix S1

## Data Availability

I confirm that the RNA‐seq data are available on the Gene Expression Omnibus (GEO) platform and the accession number is GSE190542. The accession number can be found in the supplemental data section.
