# Supplementary material for: Germline ERCC excision repair 6 like 2 ( ERCC6L2 ) mutations lead to impaired erythropoiesis and reshaping of the bone marrow microenvironment
Source: Br J Haematol. 2022 Sep 26;199(5):754–64. doi: 10.1111/bjh.18466 (PMC9828415; doi:10.1111/bjh.18466)
Supplement: Supplementary file 1 — Appendix S1 [file BJH-199-754-s001.docx]

**Germline *ERCC6L2* mutations lead to impaired erythropoiesis and reshaping of the bone marrow microenvironment**

Hannah Armes^1^, Findlay Bewicke-Copley^1^, Ana Rio-Machin^1^, Doriana Di Bella^2^, Céline Philippe^2^, Anna Wozniak^1^, Hemanth Tummala^3^, Jun Wang^1^, Teresa Ezponda^4^, Felipe Prosper^4,5^, Inderjeet Dokal^3^, Tom Vulliamy^3^_,_ Outi Kilpivaara^6,7,8^, Ulla Wartiovaara-Kautto^6,9^, Jude Fitzgibbon^1,#^ and Kevin Rouault-Pierre^2,#^.

1 Centre for Genomics and Computational Biology, Barts Cancer Institute, Queen Mary University of London, London, UK

2 Centre for Haemato-Oncology, Barts Cancer Institute, Queen Mary University of London, London, UK

3 Centre for Genomics and Child Health, Blizard Institute, Queen Mary University of London, London, UK

4 Área de Hemato-Oncología, CIMA Universidad de Navarra, Instituto de Investigación Sanitaria de Navarra (IDISNA), Centro de Investigación Biomédica en Red de Cáncer, CIBERONC, Pamplona, Spain

5 Clínica Universidad de Navarra, Pamplona, Spain

6 Applied Tumor Genomics Research Program, Faculty of Medicine, University of Helsinki, Helsinki, Finland

7 HUSLAB Laboratory of Genetics, HUS Diagnostic Center, Helsinki University Hospital, Helsinki, Finland

8 Department of Medical and Clinical Genetics, Medicum, Faculty of Medicine, University of Helsinki, Helsinki, Finland

9 Department of Hematology, Helsinki University Hospital Comprehensive Cancer Center, Helsinki, Finland

# Authors contributed equally

**Supplemental information**

**Experimental procedures**

**Cell purification and transduction of MSCs**

MNCs were isolated from BM by centrifugation using Ficoll-Paque PLUS (GE Healthcare Life Sciences, Buckinghamshire, UK) followed by RBC lysis. CD34^+^ cell selection was performed using EasySep™ Human CD34 Positive Selection Kit II (StemCell Technologies, BC, Canada) according to the manufacturer’s instructions. CD34^-^ cells were seeded in tissue-culture treated flasks to isolate MSCs based on plastic adherence. MSCs were cultured in MEM-α media (ThermoFisher, MA, USA) supplemented with 10% MSC-qualified FBS, nucleosides and GlutaMAX. Virus particles were added to the cells at a MOI of 10 and incubated at 37ºC, 5% CO_2_ overnight. Cells were washed, resuspended in supplemented MEM-α media and expanded for 4 days prior to FACS.

**Cell sorting**

Cell sorting was performed using a FACSAria Fusion Flow Cytometer Analyzer (BD Biosciences, CA, USA). Transduced HSPCs were stained with DAPI and monoclonal antibody against CD34. Cells were sorted based on marker gene (EGFP^+^) and CD34^+^ expression (CD34 PerCP-Cy5.5, BD Biosciences, CA, USA) and then seeded directly for downstream assays. Transduced MSCs were stained with DAPI, sorted based on EGFP^+^ expression and then seeded directly for downstream assays.

**Colony forming unit assay**

Two hundred and fifty *ERCC6L2-*KD or Scramble CD34^+^ cells, or 500 patient or healthy donor CD34^+^ cells, were seeded in 0.5 mL MethoCult Classic (#H4434 StemCell Technologies, BC, Canada) supplemented with 1% penicillin/streptomycin in a 24-well plate. Colonies were grown under hypoxic conditions (37ºC and 3% O2). Following 14 days of culture, colonies were scored and imaged using a phase-contrast microscope (EVOS™).

**Culture of primary MSCs**

MSCs were used at low passage (lower than 5^th^) and cultured in expansion media (MEM-α media supplemented with 10% MSC-qualified FBS, nucleosides and GlutaMAX). Medium was refreshed every 2-3 days for a total of 21 days. Osteoblast differentiation was induced using a Human Mesenchymal Stem Cell Osteogenic Differentiation Medium BulletKit^TM^ (Lonza, Basel, Switzerland) according to the manufacturer’s instructions and media was refreshed every 3 days for a total of 21 days. Mineralization of mature osteoblasts was quantified using Alizarin Red. Wells were imaged across 8 fields of view and cellular staining was quantified using ImageJ software. (1) Adipocyte differentiation was induced using a Human Mesenchymal Stem Cell Adipogenic Differentiation Medium BulletKit^TM^ (Lonza, Basel, Switzerland) according to the manufacturer’s instructions. Medium was refreshed every 2-3 days for a total of 21 days. Mature adipocytes were quantified using Oil Red O. Wells were imaged across 8 fields of view and cellular staining was quantified using ImageJ software.

**Co-culture systems**

Tissue culture plates were coated with 0.3 mg/mL rat tail collagen type I and seeded with 2.5 x 10­^4^ MSCs per well of a 12-well plate. After 3 days, MEM-a media was replaced with 1 mL Myelocult H5100 (Stem Cell Technologies, BC, Canada) and 2.0 x 10^3^ HSPCs were seeded onto the MSCs. A half media change was performed every 3-4 days. After 5 weeks, HSPCs were isolated and sorted based on CD45^+^ (CD45 PE/Cy7, BD Biosciences, CA, USA) and CD34^+^ (CD34 PerCP-Cy5.5, BD Biosciences, CA, USA) expression and then seeded in a colony forming unit assay.

**Flow cytometry analysis**

Flow cytometry analysis was performed using an LSRFortessa Analyzer (BD Biosciences, CA, USA). HSPCs cultured in erythroid differentiation conditions were immunophenotyped using monoclonal antibodies specific for human antigens (CD71 PE, CD235a APC/Cy7, Biolegend, UK). Apoptotic cells were stained with Annexin-V Alexa Fluor 647 (Biolegend, UK) and DAPI. HSPC DNA content was measured by fixing cells in 70% ice-cold ethanol for 30 minutes and then staining with DAPI (2 µg/mL). All data were analysed using FlowJo software (BD Biosciences, CA, USA).

**Reverse transcriptase and quantitative PCR**

Total RNA was extracted from cells using an RNeasy Mini Kit (Qiagen, Hilden, Germany) and reverse-transcribed using a High-Capacity cDNA Reverse Transcription Kit (ThermoFisher, MA, USA). Quantitative PCR was performed using a TaqMan probe for *ERCC6L2* (Hs00418541_m1) and *B2M* as endogenous control (Hs00984230_m1), or using the SsoAdvanced™ Universal SYBR Green Supermix (BioRad, CA, USA) and oligonucleotide primers. All primer sequences are listed in Supplemental Table S3.

**Western blotting**

Western blot analysis was performed on 1x10^6^ knockdown OCI-AML3 cells, 1x10^6^ HSPCs cultured in erythroid media for 14 days and 1x10^6^ MSCs cultured in expansion media for 21 days. Total protein extracts (20 μg) were run on 4-12% Bis-Tris protein gels (NuPAGE) and membranes transferred via the iBlot^TM^ transfer device (Invitrogen, MA, USA). Membranes were probed using the following primary antibodies: anti-human *ERCC6L2* antibody (ab154484, Abcam, Cambridge, UK) and anti-human α-tubulin antibody (T9026, Sigma-Aldrich, MO, USA) followed by staining with horseradish peroxidase-conjugated secondary antibodies (DAKO, Glostrup, Denmark) and bands detected using ECL Plus (GE Healthcare, IL, USA).

**Giemsa stain**

Two-hundred thousand HSPCs cultured for 14 days in erythroid media were cytospun onto slides at 500 rpm for 5 minutes, fixed in ice-cold methanol for 10 minutes at 4°C and then left to air-dry. Slides were stained using a Giemsa stain kit (ab150670, Abcam, Cambridge, UK) per the manufacturer’s protocol and then mounted in synthetic resin. Slides were imaged using a NanoZoomer S60 Slide Scanner (Hamamatsu, Shizuoka, Japan).

**RNA-sequencing**

RNA-sequencing was performed on: Scramble, *ERCC6L2-*KD and patient CD34^+^ cells cultured in erythroid differentiation conditions for 14 days; Scramble and *ERCC6L2-*KD MSCs cultured for 21 days in osteogenic and adipogenic differentiation conditions; patient MSCs cultured for 14 days in osteogenic and adipogeneic differentiation conditions; and Scramble and *ERCC6L2-*KD MSCs cultured in expansion media for 21 days. RNA integrity was assessed by 4200 TapeStation (Agilent Technologies, CA, USA). Library preparation and mRNA sequencing was performed by Novogene (China) to generate 40 million paired-end 150 bp reads per sample using an Illumina instrument. FASTQ files were aligned to the human reference genome (GRCh38.p10) using Hisat2.(2) Gene level feature counting was then carried out on the bam files using the htseq-count function from the HTSeq package.(3) Differential gene expression (DGE) analyses were carried out using the DESeq2 package.(4) Gene Set Enrichment Analysis (GSEA) against KEGG and REACTOME pathways was carried out using the GSEAPreranked module on the GenePattern website, with genes ranked by logFC and the scoring method set to classic.(5) Where data from multiple batches were analysed using DESeq2 batch was included as a covariate in the analysis. Gene expression values were normalized using the variance stabilizing transformation (vst) function available in the DESeq2 package.

**Deconvolution analysis**

CIBERSORTx(6) was used to infer the proportion of late-stage erythroid precursor cells in the bulk RNA-seq erythroid samples. First, scRNA-seq data from polychromatic cells, orthochromatic cells and reticulocytes(7) was used to generate a signature for these cell types with the Create Signature Matrix module with the default parameters used. This signature was then used with the Impute Cell Fractions module to estimate the proportions of these cell types within the bulk RNA samples. Batch correction was run in S mode using the scRNA-seq matrix and the module was run in absolute mode. Functional annotation of the genes in the scRNA-seq derived signature was carried out using Database for Annotation, Visualization and Integrated Discovery (DAVID).(8)

**Patient samples**

The study was approved by the local ethics committee. All participants provided informed written consent to take part in the study.

**Statistical analysis**

Prism Version 8 software (GraphPad Inc, CA, USA) was used for statistical analysis. Data are displayed as the mean ± SEM unless otherwise stated. Statistical analysis was performed using an unpaired t-test for comparison of two groups. P values less than 0.05 were considered significant.

**Data availability**

RNA-seq data were deposited into Gene Expression Omnibus (GEO) (accession number: GSE190542).

**Supplemental Figures**


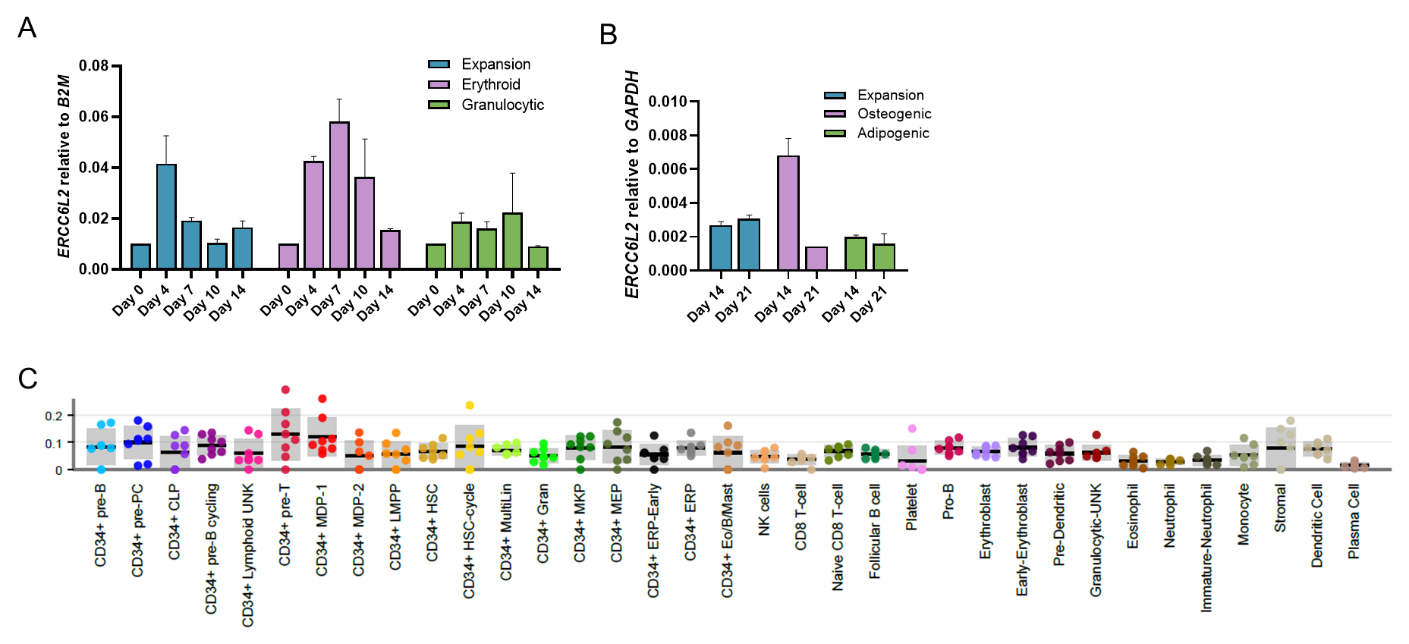


**Supplemental Figure S1. Analysis of endogenous *ERCC6L2* expression.** (A) Endogenous *ERCC6L2* expression in haematopoietic cells cultured in expansion media, erythroid differentiation or granulocytic differentiation culture conditions across multiple time points, assessed by RT-qPCR. *ERCC6L2* expression relative to *B2M.* (B) Endogenous *ERCC6L2* expression in mesenchymal stromal cells cultured in expansion media, osteogenic differentiation or adipogenic differentiation culture conditions across two time points, assessed by RT-qPCR. *ERCC6L2* expression relative to *B2M.* (C) Endogenous *ERCC6L2* expression in human bone marrow cell populations according to the Human Cell Atlas.(9)


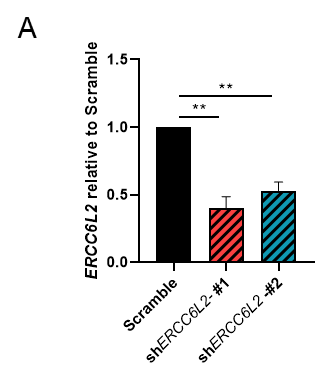


**Supplemental Figure S2. Optimization of shRNAs in the OCI-AML3 cell line.** RT-qPCR assessment of *ERCC6L2* expression relative to *B2M* in OCI-AML3 cells with sh*ERCC6L2-*#1 or sh*ERCC6L2-*#2 knockdown against Scramble control. ** p<0.01.


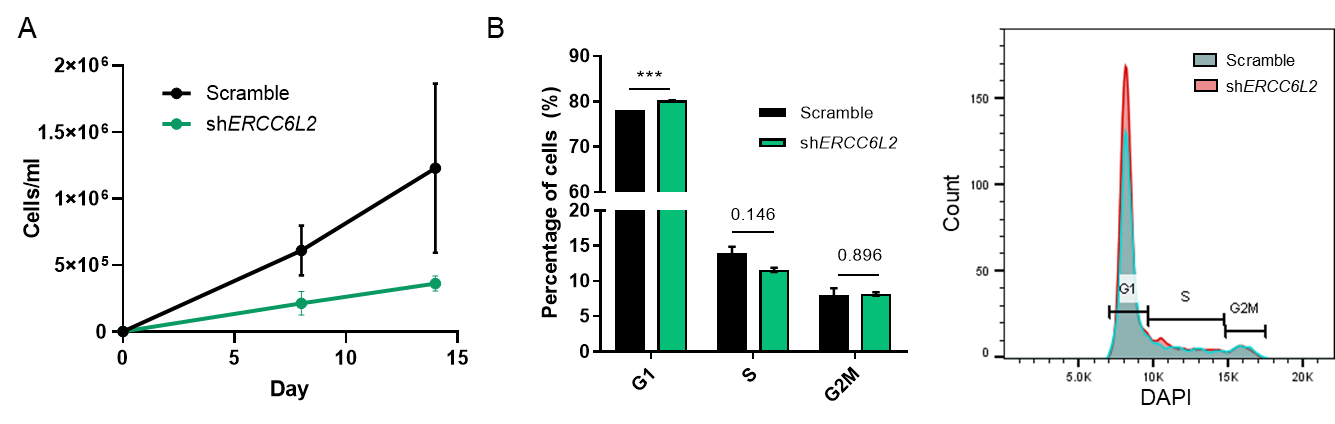


**Supplemental Figure S3. Phenotypic analysis of HSPCs with *ERCC6L2-*knockdown.** (A) Expansion rate of *ERCC6L2*-KD and Scramble HSPCs grown in erythroid media across 14 days. Data represents 3 biological repeats. (B) Cell cycle analysis of *ERCC6L2*-KD and Scramble HSPCs grown in expansion media for 3 days. Percentages of cells in G1, S and G2M phases of the cell cycle are shown. Data represents 3 biological repeats. *** p<0.001.


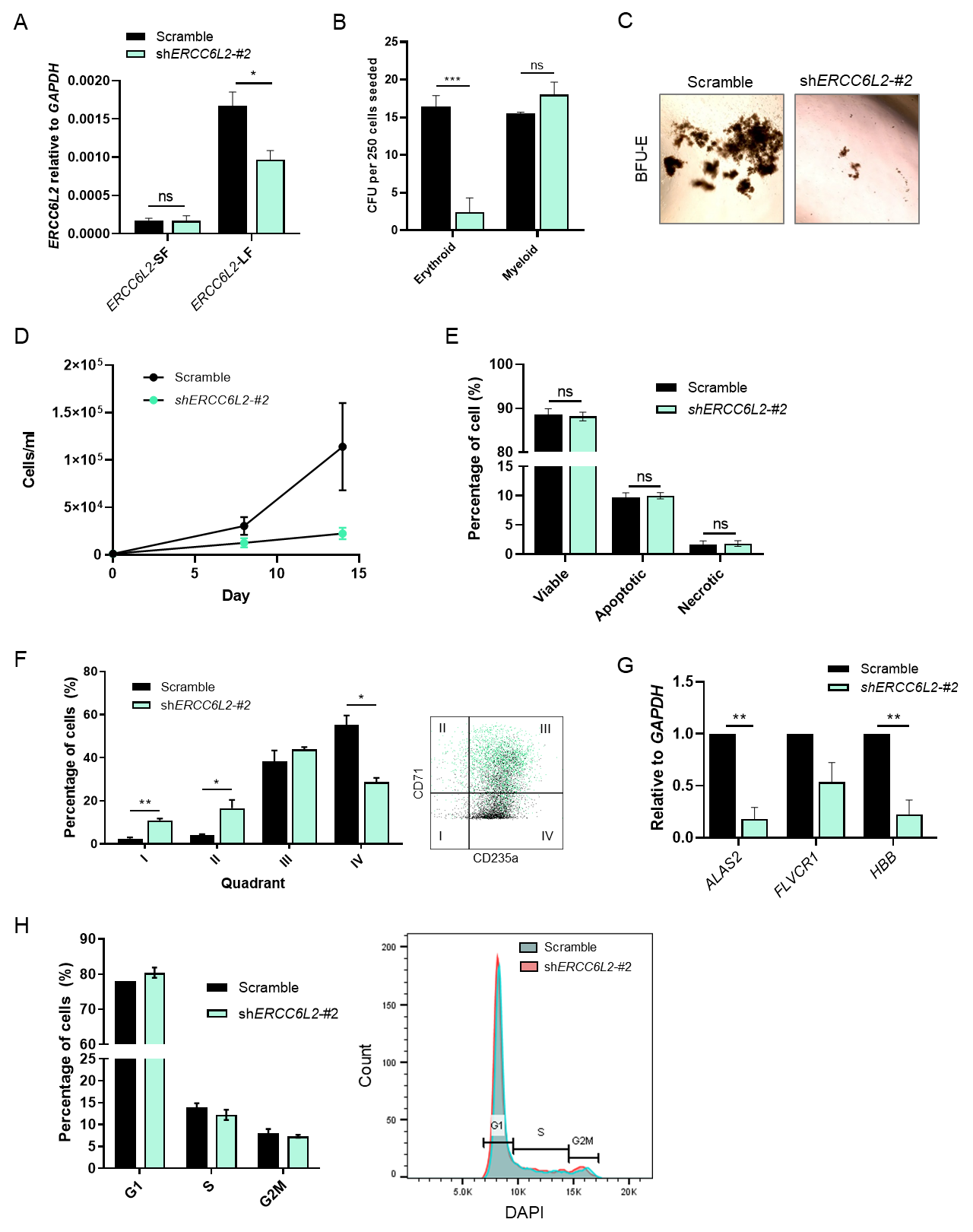


**Supplemental Figure S4. Functional validation of *ERCC6L2* knockdown with sh*ERCC6L2*-#2**. (A) RT-qPCR assessment of *ERCC6L2* short form (SF) and long form (LF) expression relative to *GAPDH* in sh*ERCC6L2-*#2 knockdown and Scramble HSPCs. Data represents 3 biological repeats. (B) CFU assay of CD34^+^ cells derived from healthy donor (UCB) and transduced with sh*ERCC6L2*-#2 or Scramble control. Colonies are separated by CFU type. (C) Representative images of morphology of BFU-E colonies derived from Scramble or sh*ERCC6L2*-#2 CD34^+^ cells. Images were captured at 4X magnification. (D) Expansion rate of sh*ERCC6L2*-#2 and Scramble HSPCs grown in erythroid media across 14 days. (E) Percentages of viable, apoptotic (Annexin-V^+^) and necrotic (DAPI^+^) HSPCs. sh*ERCC6L2*-#2 and Scramble HSPCs were grown in expansion media and analysed after 3 days of culture. (F) Immunophenotyping analysis of CD71 and CD235a expression in Scramble or sh*ERCC6L2*-#2 CD34^+^ cells cultured in erythroid media for 14 days. Representative flow plot is shown. (G) RT-qPCR of erythroid marker genes at day 14 relative to *GAPDH* in HSPCs transduced with sh*ERCC6L2*-#2. Expression of genes of interest are normalized to Scramble. (H) Cell cycle analysis of sh*ERCC6L2*-#2 and Scramble HSPCs grown in expansion media for 3 days. Percentages of cells in G1, S and G2M phases of the cell cycle are shown. Data represents 3 biological repeats. * p<0.05; ** p<0.01, *** p<0.001.


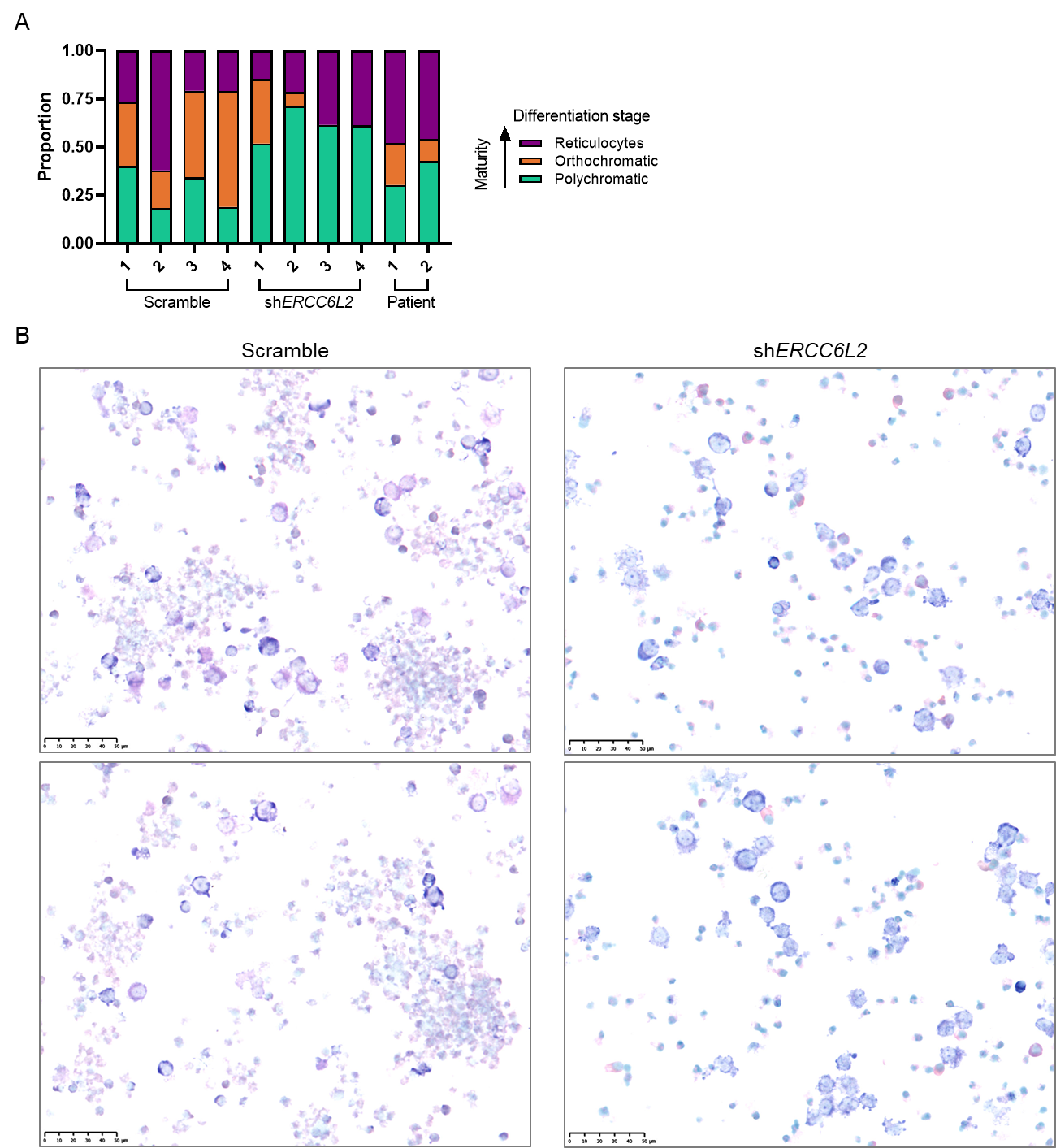


**Supplemental Figure S5. Phenotypic analysis of erythroid-committed *ERCC6L2-*deficient HSPCs.** (A) *In-silico* deconvolution analysis inferring the relative abundance of erythroid cell types within Scramble (N=4), *ERCC6L2*-KD (N=4) and patient-derived (N=2) HSPC populations which had been grown in erythroid media for 14 days and sequenced by bulk RNA-sequencing. Individual samples are shown. (B) Representative images of Giemsa-stained Scramble and *ERCC6L2-*KD HSPCs grown in erythroid media for 14 days. Scale bar represents 50 µM.

**
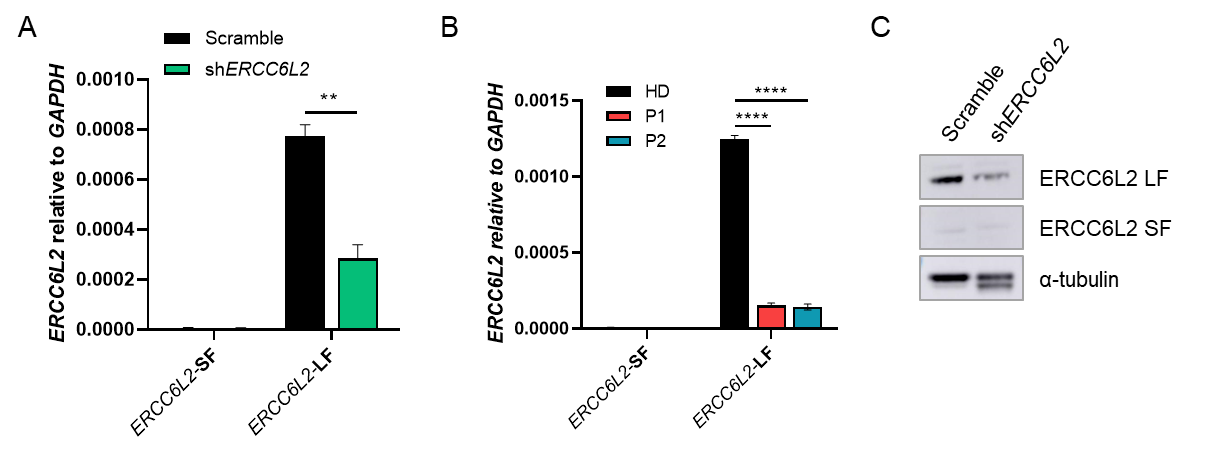
**

**Supplemental Figure S6. Analysis of *ERCC6L2* expression in *ERCC6L2-*knockdown MSCs.** (A) RT-qPCR assessment of *ERCC6L2*-SF and *ERCC6L2*-LF expression relative to *GAPDH* in *ERCC6L2*-KD and Scramble MSCs. Data represents 3 biological repeats. (B) RT-qPCR assessment of *ERCC6L2*-SF and *ERCC6L2*-LF expression relative to *GAPDH* in patient and healthy donor MSCs. (C) ERCC6L2 protein expression in Scramble and ERCC6L2-KD MSCs, analysed by Western blotting. ** p<0.01, **** p<0.0001.


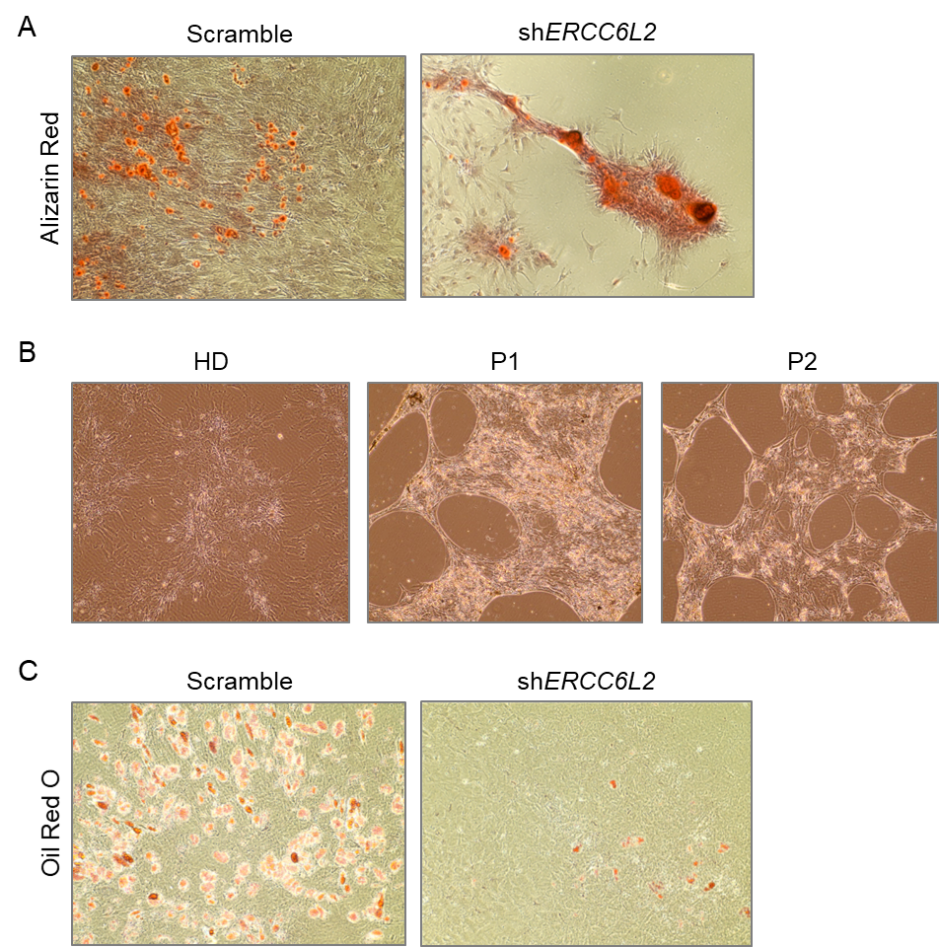


**Supplemental Figure S7. Phenotypic consequences of *ERCC6L2* loss in MSCs.** (A) Representative images of Alizarin Red staining in Scramble or *ERCC6L2-*KD MSCs following culture in osteogenic differentiation media for 21 days. (B) Representative images of patient and healthy donor MSC morphology following culture in osteogenic differentiation media for 14 days. (C) Representative images of Oil Red O staining in Scramble or *ERCC6L2-*KD MSCs following culture in adipogenic differentiation media for 21 days.

**
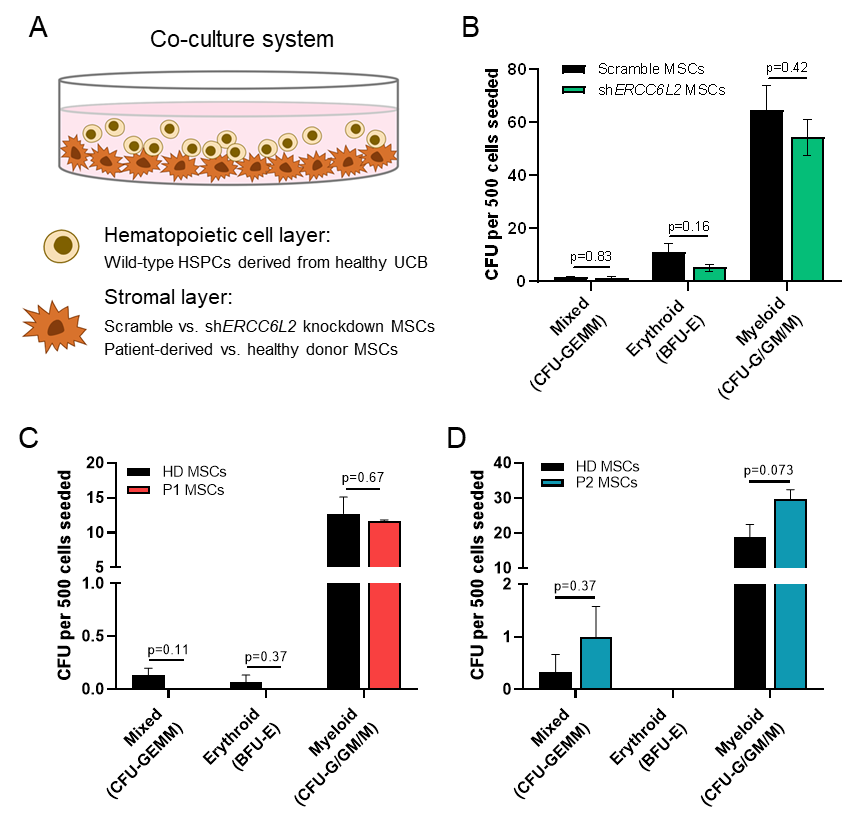
**

**Supplemental Figure S8.** **Co-culture of wild-type HSPCs upon *ERCC6L2*-deficient MSCs.** (A) Schematic of the co-culture system. (B) WT CD34^+^ cells were cultured on top of Scramble or *ERCC6L2*-KD MSCs for 5 weeks. Haematopoietic cells (CD45^+^) were then isolated and seeded in a CFU assay and the total number of colonies that formed after 2 weeks were counted. Colonies are separated by CFU type. Data represents 3 biological repeats. (C-D) WT CD34^+^ cells were cultured on top of healthy donor or patient MSCs for 5 weeks. Haematopoietic cells (CD45^+^) were then isolated and seeded in a CFU assay and the total number of colonies that formed from HSPCs after 2 weeks were counted. Data represents 3 technical repeats.

**
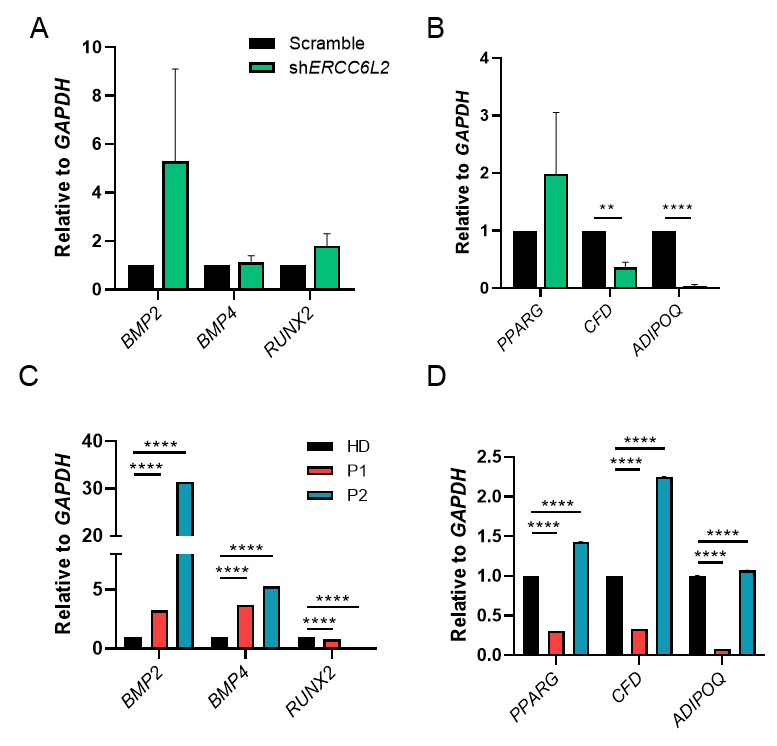
**

**Supplemental Figure S9.** **Gene expression analysis of *ERCC6L2*-deficient MSCs.**

(A) RT-qPCR analysis of osteogenic marker expression relative to *GAPDH* in knockdown cells cultured in osteogenic media for 21 days. Expression levels are normalized to Scramble. (B) RT-qPCR analysis of adipogenic marker expression relative to *GAPDH* in knockdown cells cultured in adipogenic media for 21 days. Expression levels are normalized to Scramble. (C) RT-qPCR analysis of osteogenic marker expression relative to *GAPDH* in patient-derived MSCs cultured in osteogenic media for 14 days. Expression levels are normalized to heathy donor MSCs. (D) RT-qPCR analysis of adipogenic marker expression relative to *GAPDH* in patient-derived MSCs cultured in adipogenic media for 14 days. Expression levels are normalized to heathy donor MSCs. ** p<0.01; **** p<0.0001.

**Supplemental Tables**

| **Features** | **P1** | **P2** |
| --- | --- | --- |
| Sex | Female | Female |
| Age at presentation | 8 | 36 |
| Ethnic origin | Finnish | Finnish |
| Clinical diagnosis | BMF at 8 years that spontaneously recovered at 11 years  TCP at 20 years  Puerperium cerebral vein thrombosis at 29 years  TCP with hypocellularity (5-25%) at 31 years | Grade 2 ductal mammary cancer at 36 years – treated by partial mammary resection and chemotherapy  Mild leukopenia and BM hypoplasia at 36 years |
| Germline *ERCC6L2* mutation | Homozygous c.1424del p.(Ile475ThrfsTer36) | |
| Somatic *TP53* mutation | c.659A>G p.(Tyr220Cys) 35%  c.725G>T p.(Cys242Phe) 2% | c.742C>T p.(Arg248Trp) 14%  c.818G>T p.(Arg273Leu) 3%  c.814G>A p.(Val272Met) 1.5% |
| Cytogenetics | Normal karyotype | Normal karyotype |
| Previously published | Douglas *et al*. 2019(10): patient 1438 | NA |

**Supplemental Table S1. Patient characteristics.** BMF, bone marrow failure; TCP, thrombocytopenia. Somatic *TP53* mutation variant allele frequencies (VAF) are shown.

| **Target gene** | **Reference** | **Target sequence** | **Target region** | **Reporter** |
| --- | --- | --- | --- | --- |
| *ERCC6L2* | sh*ERCC6L2-*#1 | CGGTGCCAATGTTGTTGTATT | CDS | EGFP |
| *ERCC6L2* | sh*ERCC6L2*-#2 | GAGCTGTGATTACTACCATTA | 3’UTR | EGFP |
| Non-target control | Scramble | CCTAAGGTTAAGTCGCCCTCG | NA | EGFP |

**Supplemental Table S2. shRNA target sequences.** CDS, coding DNA sequence; EGFP, enhanced green fluorescent protein; UTR, untranslated region.

| **Target gene** | **Forward (5’ to 3’)** | **Reverse (5’ to 3’)** |
| --- | --- | --- |
| *GAPDH* | CCATCACCATCTTCCAGGAG | GAGATGATGACCCTTTTGGC |
| *ERCC6L2*-SF | GGACATCCTGGAGGTGTGAACTTC | CCTTGGAGTTCATACTACCC |
| *ERCC6L2*-LF | CCTCAGATGAGAGTTTATCC | GGTTTGAGTGAATATAAGCC |
| *BMP2* | CTGCGGTGTCCTAAAGGTCG | CAACTCGAACTCGCTCAGGA |
| *BMP4* | GGAGCTTCCACCACGAAGAA | GGAAGCCCCTTTCCCAATCA |
| *RUNX2* | CAACAGAGTCATTTAAGGCTGCA | ACATGGTGTCACTGTGCTGA |
| *PPARG* | TGTGAAGGATGCAAGGGTTTCT | ATCCGCCCAAACCTGATGG |
| *CFD* | GATGTGCGCGGAGAGCAAT | CTGTCGATCCAGGCCGCATA |
| *ADIPOQ* | CGTGATGGCAGAGATGGCAC | GGTACTCCGGTTTCACCGAT |

**Supplemental Table S3. Primers.**

**Supplemental References**

1. Schneider CA, Rasband WS, Eliceiri KW. NIH Image to ImageJ: 25 years of image analysis. Nat Methods. 2012;9(7):671-5.

2. Kim D, Paggi JM, Park C, Bennett C, Salzberg SL. Graph-based genome alignment and genotyping with HISAT2 and HISAT-genotype. Nat Biotechnol. 2019;37(8):907-15.

3. Anders S, Pyl PT, Huber W. HTSeq--a Python framework to work with high-throughput sequencing data. Bioinformatics. 2015;31(2):166-9.

4. Love MI, Huber W, Anders S. Moderated estimation of fold change and dispersion for RNA-seq data with DESeq2. Genome Biol. 2014;15(12):550.

5. Subramanian A, Tamayo P, Mootha VK, Mukherjee S, Ebert BL, Gillette MA, et al. Gene set enrichment analysis: a knowledge-based approach for interpreting genome-wide expression profiles. Proc Natl Acad Sci U S A. 2005;102(43):15545-50.

6. Newman AM, Steen CB, Liu CL, Gentles AJ, Chaudhuri AA, Scherer F, et al. Determining cell type abundance and expression from bulk tissues with digital cytometry. Nat Biotechnol. 2019;37(7):773-82.

7. Berastegui N, Ainciburu M, Romero JP, Alfonso-Pierola A, Philippe C, Vilas-Zornoza A, et al. Transcriptional regulation of HSCs in Aging and MDS reveals DDIT3 as a Potential Driver of  Transformation: bioRxiv; 2021 [Available from: <https://www.biorxiv.org/content/10.1101/2021.09.08.459384v1>.

8. Huang dW, Sherman BT, Lempicki RA. Bioinformatics enrichment tools: paths toward the comprehensive functional analysis of large gene lists. Nucleic Acids Res. 2009;37(1):1-13.

9. Hay SB, Ferchen K, Chetal K, Grimes HL, Salomonis N. The Human Cell Atlas bone marrow single-cell interactive web portal. Exp Hematol. 2018;68:51-61.

10. Douglas SPM, Siipola P, Kovanen PE, Pyörälä M, Kakko S, Savolainen ER, et al. ERCC6L2 defines a novel entity within inherited acute myeloid leukemia. Blood. 2019;133(25):2724-8.
